# Schwann Cell Hamartoma Presenting as a Colonic Polyp: A Rare Case Report With a Literature Review

**DOI:** 10.7759/cureus.57674

**Published:** 2024-04-05

**Authors:** Faryal Altaf, Nismat Javed, Haider Ghazanfar, Anil Dev

**Affiliations:** 1 Internal Medicine, BronxCare Health System, New York, USA; 2 Internal Medicine, BronxCare Health System, Icahn School of Medicine at Mount Sinai, New York, USA; 3 Gastroenterology, BronxCare Health System, New York, USA

**Keywords:** colon, benign, colonoscopy, schwann cell hematoma, hamartoma, gastrointestinal

## Abstract

Mucosal Schwann cell hamartomas (MSCHs) are non-common noncancerous growths derived from Schwann cells in the peripheral nervous system, often found unexpectedly during routine colonoscopy examinations. These growths primarily occur in the colon, although they can also appear in the esophagus and are not linked to familial cancer syndromes. Diagnosis relies on specific histological characteristics and staining patterns. It is essential to distinguish MSCHs accurately since their appearance can closely resemble that of malignant tumors. Characteristically, these hamartomas test positive for S-100 protein but do not exhibit markers typical of other gastrointestinal growths, such as gastrointestinal stromal tumors (negative for KIT), leiomyomas (negative for smooth muscle actin), neurofibromas (negative for CD34), and perineuromas (negative for epithelial membrane antigen or claudin-1). This report discusses the case of a 48-year-old woman who was diagnosed with MSCH during a screening colonoscopy.

## Introduction

Mucosal Schwann cell hamartomas (MSCHs) represent a rare category of benign tumors predominantly observed in the elderly female population. These tumors, unrelated to known familial cancer syndromes, originate from Schwann cells [1,2]. The medical literature has documented only a limited number of these cases. Their identification relies on specific histological characteristics and staining patterns, underscoring the importance of precise diagnosis due to their potential resemblance to malignant growths. MSCHs are distinctively positive for S-100 protein, setting them apart from other gastrointestinal neoplasms, such as gastrointestinal stromal tumors (negative for KIT), leiomyomas (negative for smooth muscle actin), neurofibromas (negative for CD34), and perineuromas (negative for epithelial membrane antigen or claudin-1). The term MSCH was introduced into the medical literature in 2009, and these tumors have been identified in various locations within the gastrointestinal tract, including the gastroesophageal junction, gastric antrum, gallbladder, and cecum [1,3]. Schwannoma is usually asymptomatic and is an incidental finding on surveillance colonoscopies [2]. In this report, we detail the case of a 48-year-old woman diagnosed with MSCH, contributing to the body of evidence on this rare condition.

## Case presentation

A 48-year-old female with a past medical history of hypertension, asthma, and pseudotumor presented to the outpatient clinic for a screening colonoscopy for colorectal malignancies. She had a surgical history of caesarian section and bilateral tubal ligation. Her family history was significant for breast cancer in her maternal grandmother and ovarian and uterine cancer in her mother. She also reported colon cancer in her paternal side of the family at around 80 years of age. The patient was not aware of the particular details of the cancer in the family. The patient did not note any history of abdominal pain, diarrhea, weight loss, rectal bleeding, or any discomfort, and no prior endoscopic work was done. On the colonoscopy day, the patient's blood pressure was 139/87 mm Hg, her heart rate was 104 beats per minute, and her oxygen saturation (SPO_2_) was 98% on room air.

**Figure 1:**
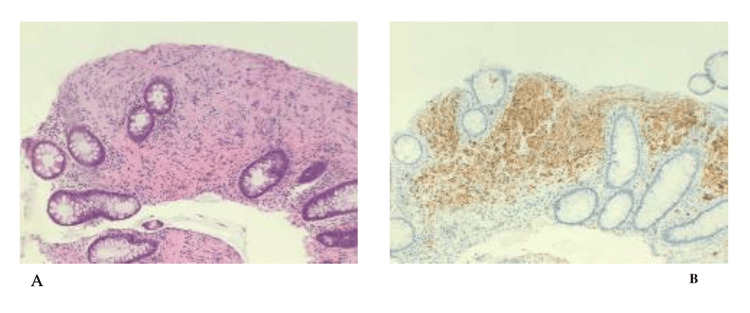
Histopathology of the colonic polyp A: Colonic mucosa with benign spindle cell lesion, consistent with a Schwann cell hamartoma. B: Immunostains show that the lesion is diffusely positive for S100.

The patient underwent a screening colonoscopy, and a 3 mm polyp was found in the sigmoid colon. The pathology of the polyp showed colonic mucosa with benign spindle cell lesion, consistent with a Schwann cell hamartoma. The immunostains show that the polyp is diffusely positive for S100 and negative for CD117, SMA, desmin, and CD34, as shown in Figure 1. Based on the colonoscopy results, the patient was seen in the gastrointestinal clinic. The patient is being followed in the primary care and gastroenterology clinics with a colonoscopy plan in one year.

## Discussion

Schwannomas are encapsulated nerve sheath tumors common in soft tissues and rare in the gastrointestinal tract [4]. They were first identified in 2009; MSCHs are distinct from schwannomas and are typically found in the colon only. However, they might be present at the rectosigmoid junction or rectum [4,5,6]. Their origin from neural cell tumors is suggested by the distribution of spindle cells and nuclear palisading [7]. The cells are usually CD34-positive and S-100 protein-positive, with S-100-negative supporting cells on their edges [8,9,10]. The lesions are typically limited to lamina propria [11]. These features are crucial to the diagnosis of the pathology. A lymphoid cuff might also be present [12]. These histological and immunohistochemical observations assist in the correct diagnosis. Although the cause of MSCHs has not been determined, these lesions are thought to result from a reactive process in areas prone to mucosal injury [12]. The differential diagnosis includes solitary/localized neuroma, schwannoma, gastrointestinal stromal tumor (GIST), colonic leiomyoma, and perineuromas.

Colorectal neurofibromas, composed of Schwann cells, fibroblasts, perineural cells, and NFP-positive axons, are one of the differential diagnoses for MSCHs [4]. They are usually associated with neurofibromatosis type 1, presenting with multiple cutaneous neurofibromas [5]. Mucosal neuromas are also one of the possible differential diagnoses but are generally found in association with multiple endocrine neoplasia syndrome type IIb (MEN2B) [4]. Ganglioneuromas are also one of the differentials and are usually associated with Cowden syndrome, juvenile polyposis syndrome, MEN2B, and NF1 [13]. Other lesions that might mimic these hamartomas include schwannomas, mucosal perineuromas, and inflammatory fibroid polyps [14]. These lesions likely represent a focus of mucosal injury, and potential risk factors for injury include chronic inflammatory diseases, such as Crohn’s disease [15]. 

Histological diagnosis to identify mucosal crypt pattern is essential in patients with Schwann cell hamartomas. As per the modified crypt pattern criteria, five types have been described: type I, round pit; type II, stellar or papillary pits; type III, tubular or small roundish pits; type IV, branch-like or gyrus-like pits; and type V, non-structural pits [16]. Usually, types I and II are considered non-neoplastic, and the rest are deemed neoplastic [16]. The pathology's treatment is primarily surgical, with resection of the polyp and subsequent follow-up of pathology [4,15,16]. Surveillance colonoscopy intervals have yet to be widely studied in the literature, and this entity has limited guidelines for surveillance colonoscopy. Reports have discussed intervals ranging from one to five years in a few cases [17,18]. To date, no complications have been documented with this entity. The literature review of Schwann cell hamartomas of the colon is mentioned in Table 1.

**Table 1:** Literature review of Schwann cell hamartomas of the colon

	Demographic (age/gender)	Clinical presentation	Indication of colonoscopy	Findings of colonoscopy	Location	Complication
1	65/Female [15]	Asymptomatic, diverticulosis	Screening	3 mm sessile polyp	Sigmoid colon	None
2	49/Male [9]	Asymptomatic history of tubular adenoma	Screening	2 mm polyp	Rectum	None
3	59/M [11]	History of ulcerative colitis, primary sclerosing cholangitis, adenomatous polyp	Screening	3 mm polyp	Sigmoid colon	None
4	67/Female [18]	History of Tubulo villous adenoma with low-grade dysplasia	Screening	3 mm sessile polyp	Rectal/30 cm away from the anal rim	None
5	40/F [5]	Asymptomatic	Screening	3 mm sessile polyp	Recto-sigmoid junction	None
6	55/Female [17]	Asymptomatic	Screening	5 mm polyp	Ascending colon	None
7	50/Female [18]	BRCA1 mutation, rectal bleeding	Diagnostic colonoscopy	2 mm polyp	Rectum	None
8	64/Male [14]	Asymptomatic	Screening	12 mm lesion	Sigmoid colon	None
9	60/Female [13]	Occult blood in stool	Diagnostic colonoscopy	5 mm sessile polyp	Recto sigmoid junction	None

## Conclusions

It is essential to keep MSCHs in mind. Although benign, their appearance can be misleading to another malignant tumor. Given that this lesion is rare, more studies are required for proper diagnosis, screening, and follow-up guidelines. Physicians, especially gastroenterologists, should remember MSCH and all other differential diagnoses associated with it while managing a patient.
